# Flavanones from* Sedum sarmentosum* Bunge Alleviate CCl_4_-Induced Liver Fibrosis in Rats by Targeting TGF-*β*1/T*β*R/Smad Pathway In Turn Inhibiting Epithelial Mesenchymal Transition

**DOI:** 10.1155/2018/3080837

**Published:** 2018-02-15

**Authors:** Yuancan Lin, Haiying Luo, Xiao Wang, Minxia Zheng, Qianxing Jin, Hongshu Chen, Peilei Pan, Junjie Zhang

**Affiliations:** ^1^The First Affiliated Hospital of Zhejiang Chinese Medical University, Hangzhou 310006, China; ^2^Hangzhou Sanatorium of People's Liberation Army, Hangzhou 310007, China; ^3^Zhejiang Chinese Medical University, Hangzhou 310053, China

## Abstract

**Objective:**

The aim of the study is to evaluate the therapeutic effects of flavanones from* Sedum sarmentosum* Bunge (FSSB) on CCl_4_-induced liver fibrosis in rats and the underlying mechanisms of action.

**Methods:**

An experimental model of liver fibrosis was established by subcutaneous injection of rats with CCl_4_ (40% v/v, 3 ml/kg) twice per week for six weeks. FSSB (100, 200, and 400 mg/kg) was intragastrically administered once per day consecutively for five weeks.

**Results:**

Our results showed that FSSB significantly attenuated CCl_4_-induced liver fibrosis as evidenced by reducing the elevated levels of serum biochemical indexes and improving the histological changes, including decreasing the elevation in serum alanine transaminase (ALT), aspartate transaminase (AST), hyaluronic acid (HA), and laminin (LN) level, reducing infiltration of inflammatory cells and collagen fibers in liver tissue. In addition, compared to the model group, FSSB markedly downregulated the protein and mRNA expression of TGF-*β*1, TGF-*β*1 receptors I and II (T*β*RI and T*β*RII), Smad2, Smad3, and Vimentin in liver tissue, at the mean time upregulating the expression of Smad7 and E-cadherin.

**Conclusions:**

The results suggest that FSSB alleviated CCl_4_-induced liver fibrosis probably through inhibition of TGF-*β*/T*β*R/Smad pathway in turn inhibiting epithelial mesenchymal transition.

## 1. Introduction

Liver fibrosis is a pathologic process caused by various risk factors, such as virus infection, alcoholism, biliary obstruction, nonalcoholic fatty liver, and inflammation. Its essence is the increased over synthesis and excessive deposition of extracellular matrix (ECM). It is believed that hepatic stellate cells (HSC) are the original cells of liver extracellular matrix formation. The activation and proliferation of HSC have been shown to contribute to liver fibrosis, which can produce a large number of ECM by expression of various signal-transducing protein. In the pathogenesis of liver fibrosis, transforming growth factor-*β*1 (TGF-*β*1) is recognized as a major profibrogenic cytokine that activates HSC [1]. In addition, TGF-*β*1 prevents decomposition of the new synthesis of ECM and increases ECM deposition, which leads to accelerating the development of liver fibrosis. It exerts its biological and pathological activities by combining with its receptors (T*β*RI and T*β*RII) and activating downstream Smads signaling pathway. With the conduction of activated Smads, TGF-*β*1 transfers its signals directly from cell surface receptors (T*β*RI and T*β*RII) into the nuclei. Moreover, Several studies have shown that TGF-*β*1/T*β*R/Smad signaling pathway mediated by TGF-*β*1 is the major information transduction pathway of liver fibrosis [2, 3]. Thus, blocking TGF-*β*1/T*β*R/Smad signaling pathway may serve as an effective strategy for preventing or treating liver fibrosis [4].

Epithelial mesenchymal transition (EMT) is a dynamic process, in which epithelial cells lose their phenotypic properties and gradually acquire mesenchymal properties [5]. It has been regarded as a vital process during liver development for a long period, and recent studies show that EMT is associated with the progress of liver fibrosis as well [6, 7]. Many cytokines and growth factors have been proved to participate in the stimulation of EMT and formation of hepatic fibrosis. For example, TGF-*β*1, a key profibrogenic mediator, is identified as an upregulator for EMT [8]. TGF-*β*1 induces EMT primarily by downstream Smads signaling pathway, which requires two types of receptor called T*β*RI and T*β*RII. Therefore, increasing expression of TGF-*β*1 and activating signal transduction mediated by TGF-*β*1 may result in the formation of EMT, which leads to liver fibrosis. If we want to inhibit EMT for curing liver fibrosis, it is an urgent task to discover some effective drugs for disrupting TGF-*β*1 production and blocking its downstream Smads signaling pathway.

*Sedum sarmentosum* Bunge (SSB), referred to as chuipencao in Chinese medicine, is one of most commonly used crude herbs in Asia, which showed hepatoprotective activity and is often used to treat acute and chronic hepatitis [9]. In previous studies, SSB had protective effects on anti-inflammation, antiaging, protecting the liver, reducing aminotransferase, antitumor, antihepatic fibrosis, and promoting the regeneration of liver cells [10–13]. Flavanones from* Sedum sarmentosum* Bunge (FSSB) are the major bioactive ingredient derived from the herbal SSB, which contains quercetin, luteolin, kaempferol, and isorhamnetin. To this day, it has been reported that FSSB can treat chronic inflammatory, prevent germs from breeding, prevent against hepatoma, and treat the acute liver injury. In addition, FSSB has the ability to inhibit bacteria proliferation of HSC, induce apoptosis of HSC, and prevent fibrosis [14, 15]. However, the pharmacological effects of FSSB on fibrosis remain debatable.

Therefore, the aim of the study is to evaluate the therapeutic effects of FSSB against CCl_4_-induced liver fibrosis and further explore the possible mechanisms of action. Our results demonstrated that FSSB played a therapeutic role in CCl_4_-induced liver fibrosis probably through inhibition of TGF-*β*1/T*β*R/Smad pathway and epithelial mesenchymal transition.

## 2. Materials and Methods

### 2.1. Preparation of FSSB and Its Quality Control

*Sedum sarmentosum* Bunge (SSB) was purchased from Huadong Medicine Co., Ltd. (Zhejiang, China) and identified by Professor Xiong Yaokang at Department of Pharmacognosy, Zhejiang Chinese Medical University. The extraction and purification of FSSB were carried out according to our previous study. Briefly, the herb was soaked and extracted twice with 80% ethanol at 80°C for 1.5 h each. After filtration, the extract solution was properly condensed and then treated by petroleum ether 3 times. Then, the treated-extract was reduced pressure distillation to recover ethanol and purified by D101 macroreticular resin and column chromatography polyamide. The extract solution was first added to D101 macroreticular resin column for adsorption for 24 h and eluted with 6-fold bed volume of ethanol (10%, 30%, 50%, and 70%) at 2 ml/min. Each elution fraction was collected and then subjected to polyamide column chromatography in order to purify further. The method was similar to D101 macroreticular resin. Finally, the eluting solution was collected and evaporated to dryness as flavonoid extracts, named FSSB.

The total flavonoids content was measured by UV spectrophotometry as described previously [16]. Briefly, using rutin as reference substance and aluminum nitrate as chromogenic agent, the flavonoid extracts were determined by colorimetry at 506 nm and calculated as rutin equivalents in terms of mg per g solid.

A Shimadzu (Japan) HPLC system, consisting of four pumps, automatic sampler, column temperature box, and UV detector, was used to determine the contents of four flavonoid compounds, quercetin, luteolin, kaempferol, and isorhamnetin. The HPLC column was a Kromasil KR-5C18 (Ф250 mm × 4.6 mm, 5 *μ*m). The mobile phase was a mixture of (A) methanol and (B) 0.1% formic acid (48 : 52, v/v); the flow rate was kept at 1.0 ml/min; the detection wavelength was 360 nm; the chromatographic column temperature was 30°C; the injection volume was 10 *μ*l. According to the retention times of the sample peaks compared with those of the true reference standard, four compounds were identified. The content of each compound in FSSB was determined by using external standard method.

### 2.2. Animals and Treatment

Male SD rats (*n* = 60, 6 to 8 weeks of age, 230–250 g) were purchased from the Shanghai Sipur-Bikai Experimental Animal Ltd (Quality certificate number: SCXK (Hu) 2013-0016). The rats were housed in the Laboratory Animal Center of Zhejiang Chinese Medicine University.

The total rats were randomly divided into six groups (*n* = 10 in each group): the normal control group, the model group, the FSSB 100 mg/kg group, the FSSB 200 mg/kg group, the FSSB 400 mg/kg group, and the silymarin 200 mg/kg group. For our experiment, the dosing regimen of FSSB was chosen based on the previous studies concerning antihepatic fibrosis of FSSB [14]. The liver fibrosis rat model was induced by CCl_4_ as described previously [17, 18]. Briefly, the rats (except the normal control group) were subcutaneously injected with 3 ml/kg 40% CCl_4_ (v/v, dissolved in olive oil) twice per week for six consecutive weeks. Meanwhile, the normal control animals were injected with an equal volume of olive oil. At the end of the six-week CCl_4_ treatment, FSSB (100, 200, and 400 mg/kg) dissolved in physiological saline was intragastrically administered once per day consecutively for five weeks. The silymarin group was intragastrically treated with silymarin as a positive group. The rats in the normal control group and the model group received the equal volume of physiological saline daily at the same time. After the eleven-week experimentation period, the rats were anesthetized with pentobarbital sodium and then sacrificed. Biochemical indicators samples were collected and liver specimens were dissected out from all animals. A portion of liver tissue was fixed in 4.5% buffered formalin for histopathological analysis, and another the other portion of the liver tissue was stored at −80°C for the measurements of mRNA and protein.

### 2.3. Biochemical Measurements in Blood

Plasma alanine transaminase (ALT) and aspartate transaminase (AST) levels were measured by using an automatic biochemical analyzer. And the levels of hyaluronic acid (HA) and laminin (LN) were determined according to the manufacturer**'**s instructions. All serum biochemical indexes were detected in the biochemistry laboratory of the First Affiliated Hospital of Zhejiang Chinese Medical University.

### 2.4. Histological Analysis

The liver tissues excised from each animal were fixed in buffered formalin (4.5% solution) for at least 24 h. After embedding in paraffin, the fixed tissues were cut into 5 mm thick sections and then stained with hematoxylin and eosin (HE) and Masson's trichrome to evaluate the degree of liver fibrosis.

### 2.5. Quantitative Polymerase Chain Reaction (PCR)

The mRNA expression of TGF-*β*1, TGF-*β*1 receptor I (T*β*RI), TGF-*β*1 receptor II (T*β*RII), Smad2, 3, 7, E-cadherin, and Vimentin was checked by Real time PCR, which was performed as described previously [19, 20]. Total RNA was isolated from liver tissue samples using RNA isoPlus, and then mRNA was reversely transcribed into cDNA according to the reagent instructions. The primer sequences (Table 1) were synthesized by Shanghai Jierui company. Using GAPDH as internal reference, relative mRNA expression levels were determined according to the 2^−ΔΔCt^ method [21].

### 2.6. Western Blotting Analysis

Liver tissue (100 mg) from the experimental rats was placed in the EP tube which contains 1 ml RIPA lysate and homogenized sufficiently on ice. The debris of tissue was removed by centrifugation at 12,000 rpm at 4°C for 10 min. Proteins extracted from the remaining supernatant were analyzed through using a Bradford assay kit. The total proteins were resolved in sodium dodecyl sulfate-polyacrylamide gel electrophoresis (SDS-PAGE) and transferred onto polyvinylidene (PVDF) membrane. After blocking with 5% nonfat milk for 60 minutes, the membranes were subsequently incubated with appropriate primary antibodies overnight and horseradish peroxidase-conjugated secondary antibodies. Primary antibodies against TGF-*β*1, T*β*RI, T*β*RII, Smad2, 3, 7, E-cadherin, and Vimentin were obtained from Abcam, and a primary antibody against *β*-actin was obtained from Santa Cruz. After washing the membrane, the density of the immunoreactive bands was further analyzed by densitometry using an enhanced chemiluminescence detection system.

### 2.7. Statistical Analysis

All statistical analysis was performed with SPSS software (Version 17.0), and all data were showed as means ± SD. Differences between two groups were assessed for statistical significance by using a one-way analysis of variance. *P* < 0.01 or *P* < 0.05 was considered statistically significant.

## 3. Results

### 3.1. Quality Evaluation of FSSB

**Figure 1:**
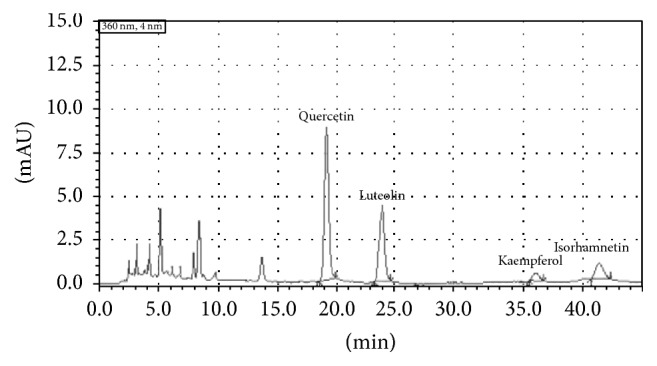
HPLC analysis of four flavonoid components in FSSB.

The content of total flavonoids in FSSB, calculated as rutin equivalent, was 701.21 mg/g. By HPLC analysis, as shown in Figure 1, the contents of quercetin, luteolin, kaempferol, and isorhamnetin in FSSB were 128.32 mg/g, 86.23 mg/g, 38.75 mg/g, and 45.67 mg/g, respectively.

### 3.2. FSSB Alleviates CCl_4_-Induced Functional and Histological Damage

After CCl_4_ injection twice a week for six weeks, the rats experienced serious liver damage, as indicated by increased serum biochemical indexes, including ALT, AST, HA, and LN (Table 2). However, the concomitant FSSB treatment had a therapeutic effect against CCl_4_-induced injury, as evidenced by reducing these elevated levels of ALT, AST, HA, and LN relative to the model control in a dose-dependent manner (Table 2).

**Figure 2:**
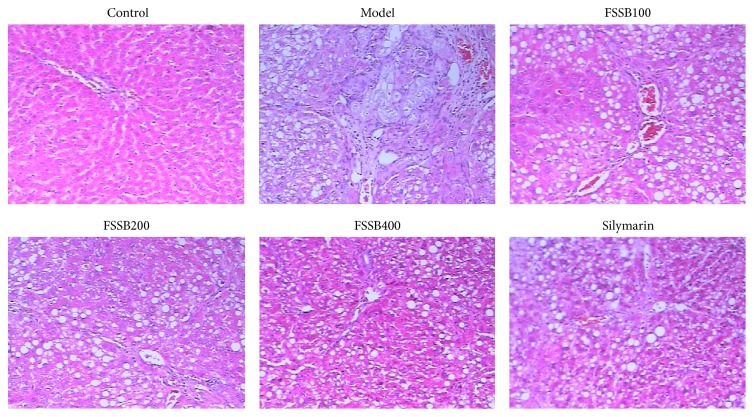
Effect of FSSB on liver histopathology stained with hematoxylin and eosin (HE).

**Figure 3:**
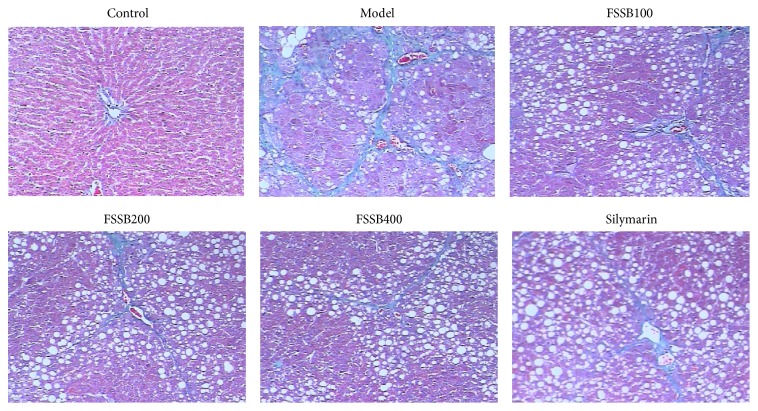
Effect of FSSB on liver histopathology stained with Masson's trichrome.

The liver sections were stained with HE and Masson's trichrome for histological evaluation of liver fibrosis. As shown in Figures 2 and 3, the hepatic lobules of normal control group were arranged in order and ranged radially around the central vein. There was no degeneration, necrosis, and collagen proliferation in liver tissue. Following CCl_4_ administration for six weeks, severe liver injury and pathological changes were observed relative to the normal control, including the disruption of hepatic lobule structure, hepatocyte necrosis, the disorder of hepatic cord arrangement, infiltration of inflammatory cells, massive collagen proliferation, pseudolobular formation, and widely distributed fibers (Figures 2 and 3). And yet, compared to the model group, FSSB treatment markedly prevented histological damage and ameliorated the pathological changes, as shown in Figures 2 and 3, suggesting that FSSB plays a therapeutic role in CCl_4_-induced liver fibrosis.

### 3.3. FSSB Protects against CCl_4_-Induced Liver Fibrosis by Targeting TGF-*β*1/T*β*R/Smad Pathway

**Figure 4:**
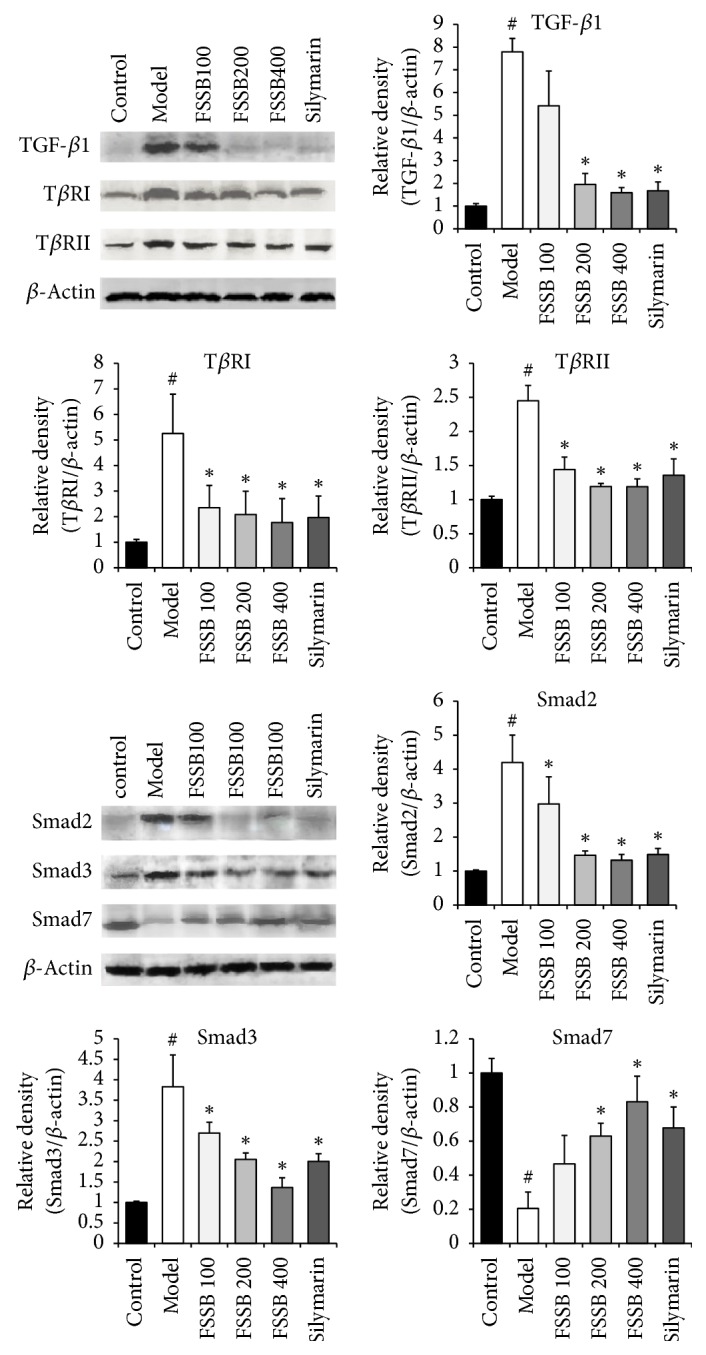
Effect of FSSB treatment on protein expression levels of hepatic TGF-*β*1/T*β*R/Smad. ^#^*P* < 0.01 compared to the normal control group; ^*∗*^*P* < 0.05, compared to the model group.

**Figure 5:**
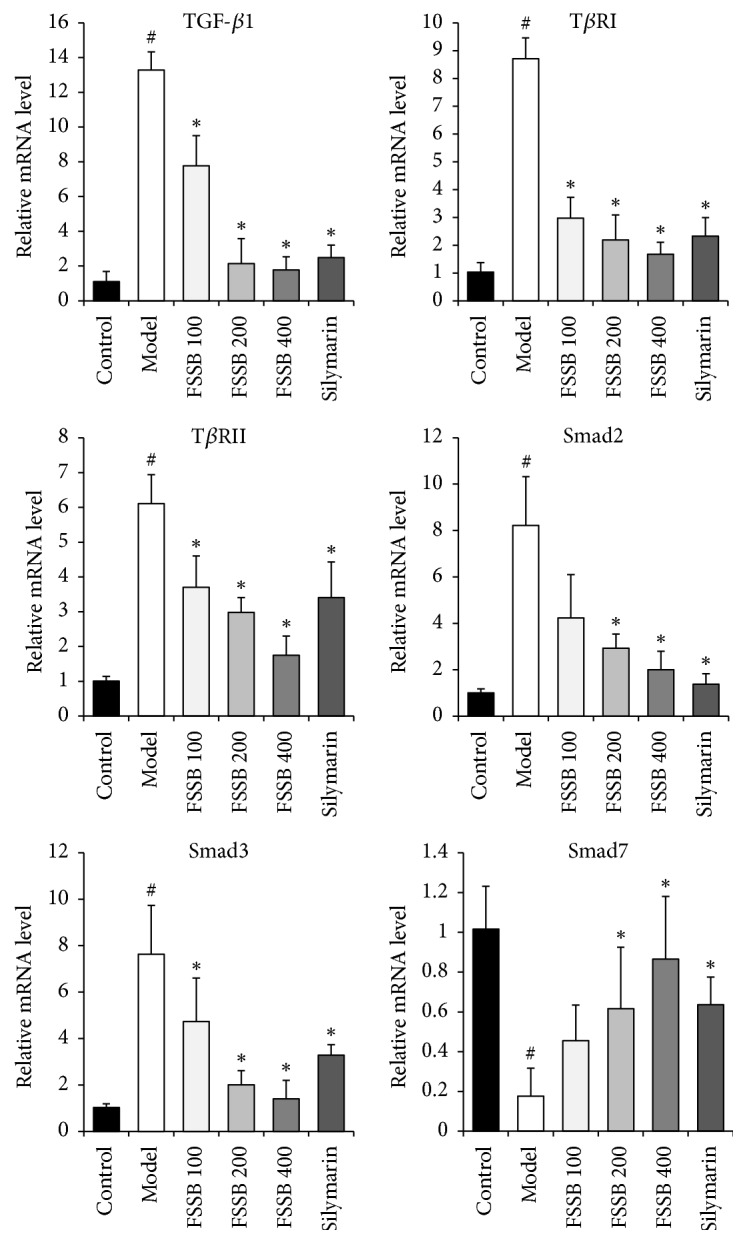
Effect of FSSB treatment on mRNA expression levels of hepatic TGF-*β*1/T*β*R/Smad. ^#^*P* < 0.01, compared to the normal control group; ^*∗*^*P* < 0.05, compared to the model group.

Since TGF-*β*1/T*β*R/Smad pathway plays an important part in the progress of liver fibrosis, we first analyzed the protein expression levels of TGF-*β*1/T*β*R/Smad in experimental rat liver tissue. According to the Western blotting results, CCl_4_ powerfully enhanced the protein expression of TGF-*β*1 and its receptors (T*β*RI and T*β*RII) in liver tissue from the model group rats, and a significant decrease in hepatic Smad7 (Figure 4). Moreover, it was observed that both Smad2 and Smad3 protein expressions in liver of rat from the model group were increased markedly relative to normal control group (Figure 4). After FSSB treatment for five weeks, the result showed that FSSB could block the increase of TGF-*β*1, T*β*RI, T*β*RII, Smad2, and Smad3 protein expression, at the mean time upregulating the protein expression of Smad7 (Figure 4). Additionally, the mRNA levels of above cytokines were measured by RT-PCR. Changes in the mRNA expression levels of TGF-*β*1/T*β*R/Smad were similar to their protein expression levels (Figure 5). The above results clearly indicate that FSSB protected against CCl_4_-induced liver fibrosis by targeting TGF-*β*1/T*β*R/Smad pathway.

### 3.4. FSSB Inhibits Epithelial Mesenchymal Transition in Chronic Liver Fibrosis due to CCl_4_

**Figure 6:**
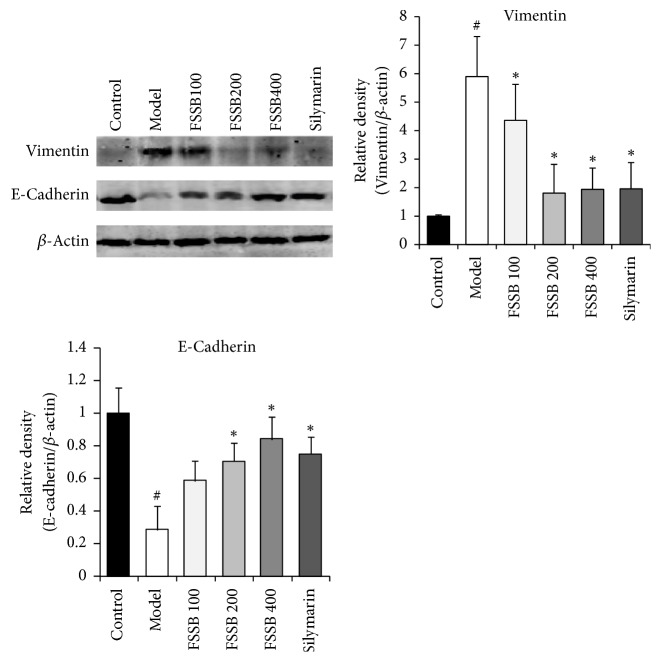
Effect of FSSB treatment on protein expression levels of hepatic E-cadherin and Vimentin. ^#^*P* < 0.01, compared to the normal control group; ^*∗*^*P* < 0.05, compared to the model group.

**Figure 7:**
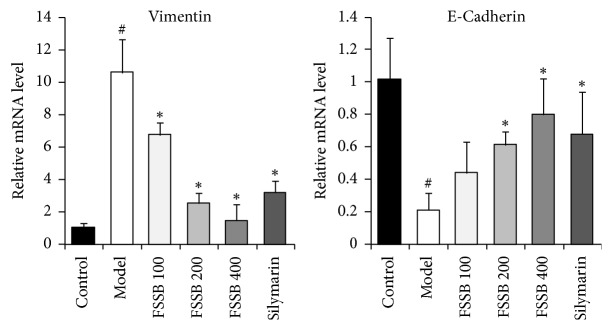
Effect of FSSB treatment on mRNA expression levels of hepatic E-cadherin and Vimentin. ^#^*P* < 0.01, compared to the normal control group; ^*∗*^*P* < 0.05, compared to the model group.

To investigate whether the antifibrotic effect of FSSB was mediated via inhibition of epithelial mesenchymal transition (EMT) in hepatocytes, we measured the levels of E-cadherin and Vimentin in liver tissue by Western blotting and RT-PCR assay. As shown in Figures 6 and 7, compared to the normal control group, CCl_4_ administration resulted in a significant increase in the expression of Vimentin and decrease in the expression of E-cadherin. Following FSSB treatment, it clearly suggested that FSSB markedly attenuated the protein expression of these two cytokines compared with the model group. As evident from Figure 6, FSSB had the ability to suppress Vimentin protein expression in liver tissues, meanwhile upregulating the protein expression of E-cadherin in dose-dependent manner. Moreover, the effect of FSSB on the E-cadherin and Vimentin mRNA expression was similar to their protein expression levels (Figure 7). As a result, it was revealed that FSSB had a strong inhibitory property on the EMT in the progress of liver fibrosis.

## 4. Discussion

As a common disease, liver fibrosis is considered as an outcome of chronic liver injury for a long period, which will result in cirrhosis and liver failure in advanced stages [22]. As we have known, liver fibrosis has become a worldwide disease which seriously threatens people's health, but it is reversible. However, there is lack of ideal drugs as the antifibrotic agents for curing hepatic fibrosis at present [23]. Therefore, it is an urgent task to discover some effective drugs for preventing or treating liver fibrosis and investigate the mechanism of action against the disease as well. Recently, Several studies clearly demonstrated that some plant-derived drugs such as curcumin, silymarin, glycyrrhizin, and notoginsenoside had beneficial effects on preventing or treating liver fibrosis via different mechanisms [24–27]. Although it is reported that flavanones derived from SSB can prevent liver fibrosis to some extent due to their anti-inflammatory property, the treatment of hepatic fibrosis is not clear. To this day, little is known about the relationship between T*β*R and EMT in the study of the mechanism against liver fibrosis. In the present study, we reported that FSSB had therapeutic effects on CCl_4_-induced liver fibrosis and its underlying mechanism of action. The result indicated that FSSB modulated TGF-*β*1/T*β*R/Smad signaling pathway in turn inhibiting EMT against liver fibrosis during the progress of the disease.

Liver fibrosis induced by CCl_4_ is a classical model of chemical liver damage, which is widely used to study the liver function and histology changes of liver fibrosis and cirrhosis [28]. In this study, animals were subcutaneously injected with CCl_4_ to establish the model of liver fibrosis, according to Domitrović and Jakovac [29]. The histopathological analysis showed that CCl_4_ caused obvious hepatic steatosis, necrosis, infiltration of inflammatory cells, massive collagen proliferation, pseudolobular formation, and widely distributed fibers. Our results indicate that intragastrical administration of FSSB once per day consecutively for five weeks can significantly alleviate the symptoms in the hepatopathy rats including improving hepatic steatosis, alleviating lobular inflammatory cells infiltration, and decreasing massive collagen proliferation. It is generally accepted that ALT and AST are regarded as the most sensitive indicators of hepatic cell damage, and HA and LN are related to activation of HSC and formation of hepatic fibrosis. In this context, the serum levels of ALT, AST, HA, and LN were pathologically elevated due to the hepatotoxicity of CCl_4_. However, the concomitant FSSB treatment reduced the elevated levels of these serum biochemical indexes relative to the model control in a dose-dependent manner. These results confirm that FSSB played a protective role in CCl_4_-induced liver fibrosis in this rat model.

In the process of liver fibrosis, TGF-*β*1 is considered as a key profibrotic cytokine [2]. It is the most potent fibrogenesis factor with certain effect on promoting ECM by activating HSC. As we have known so far, TGF-*β*1 must combine with its receptors (T*β*RI and T*β*RII) and rely on downstream Smads signaling pathway to exert its biological and pathological activities. In the classic TGF-*β*1 signaling pathway, activated TGF-*β*1 binding to the TGF-*β* type II receptor (T*β*RII) recruits the TGF-*β* type I receptor (T*β*RI), which leads to the formation of the compound TGF-*β*1/T*β*RII/T*β*RI. On the other hand, this compound subsequently recruits and activates Smad2 and Smad3. The activated Smad2 and Smad3 bind with Smad4 and together they allow translocation of the complex into the nucleus to regulate expression of the target genes. Moreover, as an inhibitor of TGF-*β*1/Smad signaling, Smad7 inhibits Smad2 phosphorylation and abolishes TGF-*β*1 response [30]. Furthermore, TGF-*β*1 signals directly migrate from cell surface receptors (T*β*R) into the nuclei with the guide of activated Smads during the development of hepatic fibrosis.

Increasing evidence shows that TGF-*β*1/T*β*R/Smad signaling pathway is regarded as a potential antifibrosis target against hepatic fibrosis induced by CCl_4_: TGF-*β*1, T*β*RI, T*β*RII, Smad2, and Smad3 are highly up-regulated in the form of either transcript or protein in the fibrotic tissues of animal models or human samples, while the expression of smad7 is decreased. It is generally accepted that TGF-*β*1/T*β*R/Smad signaling pathway plays vital roles in the development of hepatic fibrosis. Our current results demonstrated that FSSB markedly downregulated the levels of T*β*RI, T*β*RII, TGF-*β*1, Smad2, and Smad3, as well as up-regulated smad7 expression. All these findings confirmed that FSSB protected against liver fibrosis induced by CCl_4_ probably through inhibition of TGF-*β*1/T*β*R/Smad pathway.

As we have known, EMT has been regarded as a crucial mechanism in the progress of hepatic fibrosis. Recent studies have demonstrated that ECM is not the only factor leading to liver fibrosis; epithelial cells can be capable of transferring and expressing typical markers of fibroblasts by EMT and then participate in the formation of liver fibrosis [31]. It has been reported that certain adult hepatocyte types have the ability to undergo EMT in vivo and excessive EMT during liver repair can cause liver fibrosis [32]. EMT can be further determined by verifying epithelial morphology through the cellular protein expression of E-cadherin, and by examining the changes of Vimentin (markers of the mesenchymal phenotype) [33]. Thus, inhibiting E-cadherin expression and increasing Vimentin level have been identified as a hallmark of EMT [34].

So far, several signal pathways have been proved to induce EMT: TGF-*β*1/T*β*R/Smad pathway, Wnt pathway, Notch pathway, and PI3K/Akt pathway. Among these pathways, TGF-*β*1/T*β*R/Smad pathway is especially related to EMT through its ability to increase Vimentin expression and suppress E-cadherin level. It is recognized that TGF-*β*1 is the most important upregulator of EMT because it can bring about the EMT in many epithelial cells [35]. Besides, EMT in tumor cells such as colon cancer cells, skin cancer cells, and breast cancer cells as well can be reversed via lowering the expression of T*β*R. Targeted therapy that inhibits the expression of T*β*RI might have the ability to block EMT by promoting the formation of epithelial phenotype and reducing mesenchymal differentiation. As reported before, Smads were also associated with EMT [36]. It is therefore interesting that the high levels of Smads, such as Smad2, Smad3, and Smad4, can promote the formation of EMT in mammary epithelial cells, while Smad7 is considered as a negative regulator of EMT induced by TGF-*β*1. In this study, our findings demonstrated that FSSB suppressed the hepatocyte EMT in CCl_4_-induced fibrosis in vivo. Administration of FSSB significantly increased the expression of epithelial marker E-cadherin and decreased mesenchymal marker Vimentin.

## 5. Conclusion

In conclusion, this study revealed that FSSB significantly attenuated CCl_4_-induced liver fibrosis. The detailed molecular mechanisms of action may be its strong inhibitory property on activation of TGF-*β*1/T*β*R/Smad signaling, thereby inhibiting the EMT in the progress of liver fibrosis. The findings from our investigation indicate that FSSB may be a potential novel therapeutic drug for treating liver fibrosis.

## Figures and Tables

**Table 1:** Primers used for the qRT-PCR study.

Gene	Forward primer (5′-3′)	Reverse primer (5′-3′)
T*β*RI	GATACAAGGGAGTCAAGTT	GAGATAGGTCATGGCTAA
T*β*RII	GAGTGAAGCCGTGGTAGGT	TGAGAAGCCGCAGGAAGT
TGF-*β*1	CTGCTGACCCCCACTGATA	CTGTATTCCGTCTCCTTGGTCC
Smad2	TACCACTCTCTCCCCTGTCAAT	GCAAACCTAAGCAGAACCTCTC
Smad3	TGGCTACCTGAGTGAAGATGG	AGTTATTGTGTGCTGGGGACA
Smad7	GATACCCGATGGATTTTCTCAA	AGGGCTCTTGGACACAGTAGAG
E-Cadherin	TTTGGAAGCTTTGGCTGAGT	GTTGGCCAGTCACCTGAAAT
Vimentin	AAGCAGGAGTCCACTGAGTA	GTATCAACCA GAGGGAGTGA
GAPHD	GGCACAGTCAAGGCTGAGAATG	ATGGTGGTGAAGACGCCAGTA

**Table 2:** Effect of FSSB on serum activities of AST, ALT, HA, and LN in CCl_4_-induced liver fibrosis rats.

Groups	AST (IU·L^−1^)	ALT (IU·L^−1^)	HA (ng·ml^−1^)	LN (ng·ml^−1^)
Control	229.08 ± 50.07	58.04 ± 16.44	68.69 ± 15.97	50.94 ± 16.50
Model	485.63 ± 182.11^#^	361.05 ± 246.47^#^	277.21 ± 110.69^#^	72.13 ± 8.62^#^
FSSB 100	310.49 ± 100.14^**∗**^	205.28 ± 46.89	182.06 ± 38.47^**∗**^	57.98 ± 13.06^**∗**^
FSSB 200	267.90 ± 68.33^**∗**^	181.65 ± 58.29^**∗**^	161.79 ± 51.54^**∗∗**^	53.40 ± 9.00^**∗**^
FSSB 400	227.05 ± 78.76^**∗**^	171.34 ± 88.48^**∗**^	142.59 ± 67.57^**∗∗**^	49.30 ± 7.41^**∗**^
Silymarin	254.40 ± 50.32^**∗**^	180.35 ± 54.32^**∗**^	145.83 ± 75.23^**∗∗**^	51.82 ± 9.20^**∗**^

Control: the normal control group; model: the model group; FSSB 100: the FSSB 100 mg/kg group; FSSB 200: the FSSB 200 mg/kg group; FSSB 400: the FSSB 400 mg/kg group; silymarin: the silymarin 200 mg/kg group (the same as below). ^#^*P* < 0.01, compared to the normal control group; ^**∗**^*P* < 0.05, ^**∗∗**^*P* < 0.01, compared to the model group.

## References

[1] Friedman S. L. (2008). Mechanisms of hepatic fibrogenesis. *Gastroenterology*.

[2] Breitkopf K., Godoy P., Ciuclan L., Singer M. V., Dooley S. (2006). TGF-*β*/Smad signaling in the injured liver. *Zeitschrift für Gastroenterologie*.

[3] Shen B., Yang D. M., Meng X. Y. (2007). Intracellular signal transduction in the hepatic stellate cell during liver fibrosis. *International Journal of Internal Medicine*.

[4] Sun X. N., Lou G. Q., Wang X. K. (2007). Effects of IFN-*γ* on expression of smad3 mrna in liver tissue of rats with hepatic fibrosis. *Journal of Medical Research*.

[5] Maheswaran T., Rushbrook S. M. (2012). Epithelial-mesenchymal transition and the liver: Role in hepatocellular carcinoma and liver fibrosis. *Journal of Gastroenterology and Hepatology*.

[6] López-Nouoa J. M., Nieto M. A. (2009). Inflammation and EMT: an alliance towards organ fibrosis and cancer progression. *EMBO Molecular Medicine*.

[7] Kalluri R., Weinberg R. A. (2009). The basics of epithelial-mesenchymal transition. *The Journal of Clinical Investigation*.

[8] Zavadil J., Böttinger E. P. (2005). TGF-*β* and epithelial-to-mesenchymal transitions. *Oncogene*.

[9] Dong Y. N., Chen Y. Y., Zhang F. Y., Chen J. J. (2014). Sedum sarmentosum experimental and clinical research. *Journal of Yunnan University of Traditional Chinese medicine*.

[10] Jung H. J., Kang H. J., Song Y. S., Park E. H., Kim Y. M., Lim C. J. (2008). Anti-inflammatory, anti-angiogenic and anti-nociceptive activities of Sedum sarmentosum extract. *Journal of Ethnopharmacology*.

[11] Moon S. C., Park S. C., Yeo E. J., Kwak C. S. (2009). Water dropwort (ostericum sieboldii) and sedum (sedum sarmentosum) delay H_2_O_2_-induced senescence in human diploid fibroblasts. *Journal of Medicinal Food*.

[12] Liu S. H., Li R., Qiao Y. X., Chen X., Zhang N. N., Zhang J. P. (2015). Effect of Sedum Sarmentosum on acute liver injury-induced by different causes in mice. *Chinese Journal of Liver Diseases (Electronic Version)*.

[13] Huang D., Zhang W., Huang D., Wu J. (2010). Antitumor activity of the aqueous extract from sedum sarmentosum bunge in vitro. *Cancer Biotherapy and Radiopharmaceuticals*.

[14] Lin Y. C., Luo H. Y. (2015). Study on intervention effects of sedum sarmentosum total flavanones against experimental hepatic fibrosis. *Chinese Archives of Traditional Chinese Medicine*.

[15] Lin Y. C., Luo H. Y., Jin Q. X. (2015). Study on effect of total flavanones of Sedum sarmentosum on apoptosis of hepatic stellate cells and its mechanism. *China Journal of Chinese Materia Medica*.

[16] Jia R., Liu Z. R., Fu X. T. (2007). Study on the determination method of sedum sarmentosum total flavanones. *China Practical Medical*.

[17] Iredale J. P. (2007). Models of liver fibrosis: exploring the dynamic nature of inflammation and repair in a solid organ. *The Journal of Clinical Investigation*.

[18] Lin N., Chen S., Pan W., Xu L., Hu K., Xu R. (2011). NP603, a novel and potent inhibitor of FGFR1 tyrosine kinase, inhibits hepatic stellate cell proliferation and ameliorates hepatic fibrosis in rats. *American Journal of Physiology-Cell Physiology*.

[19] Huang X. P., Kang Y., Huang H., Chen X. W., Luo W. S. (2016). Effect of total flavonoids from litchi chinensis sonn on expression of TGF-*β*1 receptor and collagen in rats with liver fibrosi. *Herald of Medicine*.

[20] Li X. X., Li J., Wang S. Z., Guo C., Zhang J. P. (2014). Inhibitory effect of luteolin on hepatic Epithelial mesenchymal transition of hepatocytes in hepatic fibrosis process. *Journal of China Pharmacy*.

[21] Livak K. J., Schmittgen T. D. (2001). Analysis of relative gene expression data using real-time quantitative PCR and the 2(T)(-Delta Delta C) method. *Methods*.

[22] Ferrell L. D., Kakar S. (2011). Liver Pathology. *Demos Medical*.

[23] Muddu A. K., Guha I. N., Elsharkawy A. M., Mann D. A. (2007). Resolving fibrosis in the diseased liver: Translating the scientific promise to the clinic. *The International Journal of Biochemistry & Cell Biology*.

[24] Wang B.-E. (2000). Treatment of chronic liver diseases with traditional Chinese medicine. *Journal of Gastroenterology and Hepatology*.

[25] Wen Z., Shi Z., Feng P., Xue X., Dong K., Wang X. (2008). Modulation of energy metabolic enzyme expression in N-nitrosodiethylamine- mediated hepatocarcinogenesis by Chinese herbs, Huqi San. *BioFactors*.

[26] Wynn T. A. (2007). Common and unique mechanisms regulate fibrosis in various fibroproliferative diseases. *The Journal of Clinical Investigation*.

[27] Xu C., Liu Z. Z., Li Z. H. (2013). Effects of Hu Qi San on preventing CCl_4_-induced liver fibrosis and inhibiting hepatocytes proliferation and apoptosis in rats. *Chinese Journal of Integrated Traditional and Western Medicine on Liver Diseases*.

[28] Tsai J. H., Liu J. Y., Wu T. T. (2008). Effects of silymarin on the resolution of liver fibrosis induced by carbon tetrachloride in rats. *Journal of Viral Hepatitis*.

[29] Domitrović R., Jakovac H. (2010). Antifibrotic activity of anthocyanidin delphinidin in carbon tetrachloride-induced hepatotoxicity in mice. *Toxicology*.

[30] Dooley S., Delvoux B., Streckert M. (2001). Transforming growth factor-*β* signal transduction in hepatic stellate cells via Smad2/3 phosphorylation, a pathway that is abrogated during in vitro progression to myofibroblasts: TGF-*β* signal transduction during transdifferentiation of hepatic stellate cells. *FEBS Letters*.

[31] Thiery J. P., Acloque H., Huang R. Y. J., Nieto M. A. (2009). Epithelial-mesenchymal transitions in development and disease. *Cell*.

[32] Choi S. S., Diehl A. M. (2009). Epithelial-to-mesenchymal transitions in the liver. *Hepatology*.

[33] Ogunwobi O. O., Wang T., Zhang L., Liu C. (2012). Cyclooxygenase-2 and Akt mediate multiple growth-factor-induced epithelial-mesenchymal transition in human hepatocellular carcinoma. *Journal of Gastroenterology and Hepatology*.

[34] Huber M. A., Kraut N., Beug H. (2005). Molecular requirements for epithelial-mesenchymal transition during tumor progression. *Current Opinion in Cell Biology*.

[35] Xu J., Lamouille S., Derynck R. (2009). TGF-*β*-induced epithelial to mesenchymal transition. *Cell Research*.

[36] Valcourt U., Kowanetz M., Niimi H., Heldin C. H., Moustakas A. (2005). TGF-*β* and the Smad signaling pathway support transcriptomic reprogramming during epithelial-mesenchymal cell transition. *Molecular Biology of the Cell (MBoC)*.

